# Genetic counselling for psychiatric conditions: exploring current perceptions of family physicians and psychiatrists in Portugal

**DOI:** 10.1007/s12687-025-00774-7

**Published:** 2025-02-19

**Authors:** B. Ribeiro, I. Homem de Melo, A. Sequeira, R. Moldovan, M. Paneque

**Affiliations:** 1 ICBAS – School of Medicine and Biomedical Sciences, University of Porto, Porto, Portugal; 2 Clínica Do Quinto Andar, Porto, Portugal; 3 CRI Porto Ocidental, ICAD, Porto, Portugal; 4 CUF S. João da Madeira Clinic, São João da Madeira, Portugal; 5 Division of Evolution, Infection and Genomics, School of Biological Sciences, University of Manchester, Manchester, UK; 6 Manchester Centre for Genomic Medicine, St. Mary’s Hospital, Manchester University NHS Foundation Trust, Manchester, UK; 7 Department of Psychology, Babeș-Bolyai University, Cluj-Napoca, Romania; 8 i3S – Institute for Research and Innovation in Health, University of Porto, Porto, Portugal; 9 IBMC-Institute of Molecular and Cellular Biology, University of Porto, Porto, Portugal; 10 CGPP – Center for Predictive and Preventive Genetics, University of Porto, Porto, Portugal

**Keywords:** Psychiatric genetic counselling, Healthcare provision, Qualitative research

## Abstract

Genetic counselling emerged as a specialized healthcare discipline in the 1960s, and since then, various sub-specialties have developed globally, namely and more recently, psychiatric genetic counselling. This study provides a pioneering exploration of family physicians and psychiatrists’ perceptions regarding genetic counselling provision and its potentialities in the context of psychiatric illnesses in Portugal. A qualitative methodology with semi-structured interviews was used. Among the participants, five were family physicians, and six were psychiatrists. Thematic analysis revealed three themes: (1) the role of genetics in healthcare, (2) barriers to psychiatric genetic counselling implementation, and (3) perceived benefits associated with its implementation. Results show that while the importance of genetics in psychiatric disorders is acknowledged, there is low literacy about genetic counselling practice from the professional groups interviewed. Also, the availability and mainstreaming of genetic testing seem to influence how genetic counselling is perceived and utilized. There is a perceived need for training and guidelines that foster the dissemination of genetics into healthcare, specifically mental healthcare. A holistic and patient-centred approach is considered essential in managing psychiatric disorders and, by extension, in psychiatric genetic counselling, as it addresses both medical and psychosocial factors. Although psychiatrists and family physicians are keen to integrate psychiatric genetic counselling into their patients’ care, it seems that certain fundamental challenges still persist in genetic healthcare provision. Future research should contribute for a more comprehensive evaluation of the readiness for psychiatric genetic counselling implementation in the country.

## Introduction

Genomic medicine is increasingly relevant across many areas of medicine, and clinical services evolved to assist individuals dealing with complex conditions for which genetic testing became available. Genetic counselling (GC) emerged as a specialist healthcare discipline in the 1960s and, since then, we can recognize its growth and its worldwide dissemination (Ormond et al. 2018). Different sub-specialties within this discipline begun to emerge, namely and more recently, psychiatric genetic counselling (PGC).

Genetic counselling is, by definition, “a process of helping people understand and adapt to the medical, psychological, and familial implications of genetic contributions to disease” (Resta et al. 2006). GC entails holistic discussion covering both genetic and environmental factors contributing to conditions, and it aims to empower individuals to make informed, autonomous decisions that resonate with their values (Austin 2023). Over the years, in the context of various conditions, research has consistently demonstrated that GC is effective when it comes to increasing patient knowledge, empowerment and autonomy in decision-making, decreasing stigma and generating high patient satisfaction, among others (Ison et al. 2019; Madlensky et al. 2017).

According to the American Psychiatric Association, a psychiatric disorder (PD) is “a syndrome characterized by clinically significant disturbance in an individual’s cognition, emotion regulation, or behaviour that reflects a dysfunction in the psychological, biological, or developmental processes underlying mental functioning” (American Psychiatric Association 2013). PDs have complex etiopathogenetic mechanisms where both genetic predisposition and environmental factors contribute to the risk and expression of these conditions. The field of psychiatric genetics is dynamic, with ongoing research aimed at unravelling the intricate genetic and environmental factors contributing to mental health disorders (Hoehe and Morris-Rosendahl 2018; Keverne and Binder 2020; Müller 2020; Murray et al. 2021). The genetic architecture of PDs is highly polygenic and includes the full spectrum of DNA variation (Gratten et al. 2014; Smoller 2019; Wand et al. 2023). Understanding the multifactorial nature of PDs is crucial for researchers, clinicians, individuals affected by these conditions and their family.

PDs are highly prevalent, and they impact the lives of millions of people worldwide. In Portugal, the prevalence of lifetime mental disorders is above 30%, mental health disorders represent 11.7% of disease-adjusted life years lost; Portugal experiences a high prevalence of depression (7.9%), anxiety (16.5%), impulse disorders (3.5%), and substance abuse (1.6%) in comparison with other European countries (Almeida et al. 2013). Although significant prevalent, this historically undervalued community is still poorly served and there is a need to reinforce coordination between Primary Health Care and hospital care in mental health (Entidade Reguladora da Saúde 2023; Magalhães et al. 2016). In Portugal, mental health care can be provided inpatient or outpatient, in the latter case in consultations, day hospitals and psychosocial rehabilitation structures. This care is provided by public, private and social entities, with agreements with the National Health System. Psychiatrists and family physicians are the primary referral points for people with PDs, whom they mainly rely on for referrals to secondary and tertiary care as needed, including to medical genetic services.

In Portugal, GC is considered a medical act, but generally entrusted to specialists in Medical Genetics (Paneque et al. 2015). Portugal has five government-funded medical genetics hospital centres integrated into the National Health System (Ordem dos Médicos 2021). Any medical doctor can refer a patient or family to the nearest medical genetics service; in addition, relatives of registered patients can request an appointment. Some private centres and general hospitals also provide genetic testing for various diseases along with GC services (Paneque et al. 2015). Genetic counselors are not yet recognized as professionals by the competent national authorities (Lungu et al. 2024). There is an urgent need to increase the number of medical geneticists, non-medical genetic professionals and other health care professionals, aiming for a multidisciplinary and user-centred approach in the public genetic services that are currently saturated and limited in resources (Costa et al. 2022; Costa et al. 2024a, b; INSA 2022; Mendes et al. 2013; Paneque et al. 2018).

PGC is an emerging field conceptually identical to GC but more psychotherapeutically focused, seeking to help patients make personal meaning of the factors that can contribute to the development of a mental health condition that they have or that runs in their family (Austin 2020; Moldovan et al. 2019). PGC practitioners view their work through the lens of valuing the interaction as primarily existential rather than solely focused on risk specific. They prioritize addressing emotional issues that arise during these discussions. Within PGC, it is emphasized the importance of addressing misconceptions about aetiology, promoting self-management behaviours to enhance a sense of control over the illness, and of providing counselling and support around the guilt, blame, shame, fear and stigma so often attached to individuals' explanations for their illnesses (Austin 2023).

In Portugal, assessment for certain neuropsychiatric conditions, like autism spectrum disorder and intellectual and developmental disorders, at present qualify as the only PDs with enough evidence supporting genetic testing as part of standard clinical practice (Direção-Geral da Saúde 2019). Nonetheless, genetic testing in PGC do not represent a fundamental paradigm shift and it is not routinely provided (Koido et al. 2023). According to Moldovan et al. (2019), PGC in Portugal, as well as in other countries, was just emerging and family history of psychiatric disorders was only discussed in clinical genetics settings if raised by a patient during intake or if uncovered by the medical professional during a session for another indication, but it was rarely the reason for the primary referral. Additionally, PGC training was not formally or routinely offered as part of the MSc in genetic counseling nor as part of medical schools and independent continuous education courses.

Although genetic testing is often the main reason to seek or to be referred to GC, professionals have an extended scope of roles and tools that make PGC beneficial in its absence (Austin 2020; Manzini and Vears 2018). Qualitative and quantitative research studies have shown meaningful positive outcomes of PGC for individuals affected by major PDs and their family. The evidence includes research on the effects and effectiveness (Costain et al. 2014a, b; Costain et al. 2014a, b; Hippman et al. 2016; Inglis et al. 2015; Moldovan et al. 2017), on the clinical need and desire from patients and relatives (Hippman et al. 2013; Lyus 2007; Morris et al. 2018; Semaka and Austin 2019; Slomp et al. 2018), on the factors hindering its implementation (Bennett et al. 2008; Ciucă et al. 2021; Koido et al. 2023; Pinzón-Espinosa et al. 2022), and others (Austin et al. 2006; Finn et al. 2005; Inglis et al. 2017; Kendall et al. 2024; Leach et al. 2016; Zhou et al. 2014).

In an attempt to keep up with the research and evidence being produced regarding PGC around the world, and as a first approach to the theme, we meant to obtain a brief overview of the current status of PGC in Portugal from the perspectives of the primary referral points for people with PDs, using a method that enabled an exploratory framework.

This study aims to explore the current perceptions of family physicians and psychiatrists regarding GC provision and its potentialities in the context of psychiatric illnesses in Portugal. Specifically, we aim to: (i) explore the perceptions of participants regarding the role of genetics in the context of psychiatric illnesses; (ii) understand the attitudes and awareness of family physicians and psychiatrists’ regarding the need to approach the genetics/hereditability of psychiatric patients and their families; (iii) explore the potential of providing GC consultations focused on psychiatric illnesses; and (iv) identify possible barriers of GC provision in this context.

## Methodology and methods

For this exploratory study, we used a qualitative methodology with semi-structured interviews to provide a deeper and more comprehensive understanding of the topic. Thematic analysis, as the chosen method, was employed to identify, analyse, and report patterns of meaning within the qualitative data, aiming to broaden knowledge on the subject (MacFarlane et al. 2014; Wainstein et al. 2023).

### Participants and recruitment

The choice of participants for this study was purposeful and based on their role as primary referral point for individuals with psychiatric disorders. By selecting psychiatrists and family physicians, we aimed to gather insights from the medical professionals who are most likely to encounter patients with PDs and who are in positions to refer these patients to GC. These practitioners' perceptions are crucial for understanding the current status of PGC in Portugal, as they are often the first point of contact for patients seeking mental health care. Additionally, their professional experiences and interactions with patients provide valuable perspectives on the potential integration of GC into psychiatric practice.

Recruitment for the interviews was conducted through professional associations and personal networks. Several national organisations representing general and family physicians, as well as psychiatrists, were contacted to disseminate information about the study. Among these, the Portuguese Association of General and Family Medicine (APMGF) and the Portuguese Association of Child and Adolescent Psychiatry (APPIA) responded and agreed to assist with participant recruitment by sharing the study information with their members. Additionally, project members further disseminated the project through their personal networks, leveraging their professional contacts within the medical community. The study information included the researchers' contact details. Interested volunteers were invited to email the researchers expressing their interest in participating in the study, attaching the consent form (previously provided) and indicating their preferred date and time for the interview.

### Instrumentation

The semi-structured interview guide was created to cover three major subjects, as briefly shown in Table 1. The guide was designed by BR, a master’s student in genetic counselling, and MP, a senior genetic counsellor, based on existing literature while being carefully adapted to explore the topic within the unique context of its first application in Portugal. Its relevance and suitability for both professional contexts were then validated by AS (a family physician) and IHM (a psychiatrist). The interview guide was revised iteratively as interviews were completed, including adding an additional question and rephrasing of questions.

**Table 1:** Semi-structured interview guide

Subjects	Points to cover
1. Familiarity with genetics as a specialty within healthcare	• Professional experience with the medical genetics service in general • General knowledge of GC
2. Importance attributed to genetics in clinical practice in the specific context of PDs	• Professional experience with the medical genetics service in the context of PDs • Awareness around psychiatric genetics • Attitudes and level of comfort exploring the genetic/heritable component of a PD
3. Psychiatric Genetic Counselling	• Knowledge, perceptions and attributed relevance • Prospects and barriers perceived of its provision

### Data collection and analysis

BR e MP conducted all the interviews. Besides the initial informed consent that we asked participants to send, at the beginning of each interview, we inquired if there were any questions related to the project before we started. Additionally, we sought their verbal consent to begin recording the interview. This two-step consent process ensured that participants were fully informed and comfortable with the study procedures, reaffirming their willingness to participate and be recorded. At the start of each interview, participants were also informed that BR was a master’s student in genetic counselling and that the study was being conducted as part of her degree requirements. MP is an experienced researcher with a strong background in studies employing this methodology, and was present in all interviews as a facilitator. Interviews were conducted in Portuguese and recorded using Microsoft Teams, transcribed verbatim using the automated transcription option of the platform and then checked for accuracy.

For data analysis, coding was performed independently by BR and MP to ensure a diverse interpretative approach. The analyses were subsequently merged, with both researchers discussing their interpretations to integrate perspectives and minimise potential individual biases. The coding process involved an initial overview by reading and immersing in the data, followed by detailed line-by-line coding to systematically apply and group codes into themes. In this study, we adhered to the concept of Theoretical Sufficiency. This approach emphasizes achieving a robust and comprehensive understanding of the core themes and categories rather than striving for absolute saturation where no new information is discovered (Wainstein et al. 2023). By the end of our data collection, we had gathered sufficient information to construct a supported theory regarding participants' perceptions about GC in PDs. Although additional interviews might have introduced minor new insights, the central theoretical understanding was already well-established.

## Results

### Demographics

Eleven interviews were conducted and recorded, resulting in eleven transcripts. Among the participants, five were family physicians, and six were psychiatrists, comprising four specializing in adult psychiatry and two in child and adolescent psychiatry. The participants exhibited a range of ages and years of professional experience. The youngest interviewee was 33 years old with one year of specialized experience, while the oldest was 67 years old with 25 years of specialized experience in their respective fields (Table 2). Interviews ranged from 24 to 47 min. The average duration of the interviews was approximately 35 min.

**Table 2:** Participants’ medical specialty, age and years of experience

Participant	Medical specialty	Age	Years of experience
*P1*	adult psychiatrist	31–35	6–10
*P2*	adult psychiatrist	66–70	21–25
*P3*	adult psychiatrist	31–35	1–5
*P4*	adult psychiatrist	31–35	1–5
*P5*	child and adolescence psychiatrist	36–40	6–10
*P6*	child and adolescence psychiatrist	31–35	1–5
*P7*	family physician	36–40	1–5
*P8*	family physician	31–35	1–5
*P9*	family physician	46–50	21–25
*P10*	family physician	41–45	11–15
*P11*	family physician	46–50	16–20

### Themes and sub-themes

**Fig. 1:**
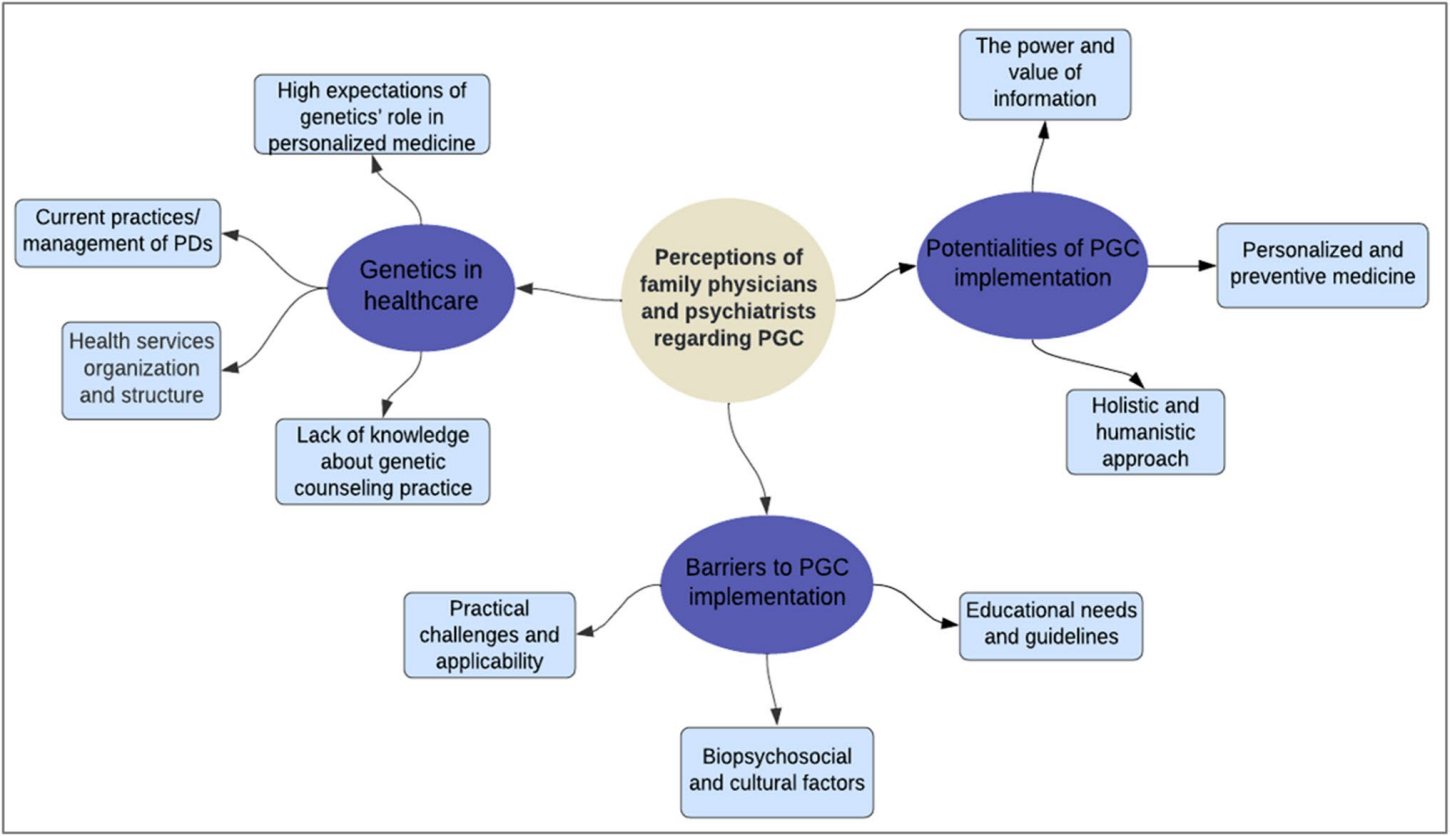
Conceptual map "Perceptions of family physicians and psychiatrists regarding PGC in Portugal"

From the collected data, three themes emerged: (1) the role of genetics in healthcare, (2) barriers to PGC implementation, and (3) perceived benefits associated with its implementation. Figure 1 illustrates the structure of these conceptual categories, including further subthemes within each theme.

### Theme 1: genetics in healthcare

Within the first theme, four sub-themes were identified. Specialists highlighted several issues related to the ***organisation and structure of health services***. The data revealed an issue with the fragmentation of care among medical specialties. The lack of integration also raises questions about which specialty should assume responsibility for PGC, and which specialty should assume the clinical responsibility of referrals in this situation.

*“Normally, as far as I know, it's rarely psychiatry that makes the referral, and if it is, it has to be from larger, more central hospitals. I think it's more often done by neurology. When there is some overlap, they also have greater coordination with the services.”* P1

The data indicates a pervasive issue of insufficient articulation between the professional groups, i.e. family physicians, psychiatrists and geneticists. Participants frequently mentioned that the communication between them (primary and secondary care providers) and the genetic services (tertiary care providers) is unidirectional and infrequent in their clinical practice.

*“The idea I have, which may be completely wrong, is that there is no established close connection with genetics, at least in the places I've been.”* P1

*“In terms of feedback, if the person is my patient, yes, they return information, especially if there is some risk or if there is any positivity. But it’s from the patient themselves who comes to the consultation and tells me if they want to. It’s not really the genetics service.”* P10

Participants emphasized the need for better-defined pathways and more robust communication channels to ensure that patients receive timely and appropriate GC and follow-up care if and when needed. There was a consensus on the need for comprehensive guidelines and robust referral networks to streamline the integration of GC into routine healthcare.

*“There has to be knowledge on the part of all of us about where to refer, what type of referral to make. (…) And it doesn't have to be guidelines in the sense of restriction, but of guidance.”* P2

Another significant sub-theme was the general ***lack of knowledge about the practice of genetic counselling***. A prevalent issue identified in the data is the widespread lack of understanding about what GC entails. Many participants associated GC primarily with the process of conducting genetic tests, often viewing it solely as a means to provide etiological clarification. Recurrence risk information also emerged as another perceived primary objective of GC.

*“So, no one, I think, with good practice, would request genetic counselling for, for example, schizophrenia, bipolar disorder, or a mood disorder, an anxiety disorder. Although we know there is a very strong genetic component in all of them.”* P2

*“I imagine there could be a phase of study, you know... of the genome in certain, in a particular clinical doubt that may exist in that person. And then based on what is found, on the yes or no of that doubt.... I presume that counselling would then be done.... which might concern, therefore, information about the risk, you know... of developing that disease; what behaviours or treatment they could undertake to reduce the risk of having that disease or of that disease becoming a problem for them? Or perhaps transmitting that disease to descendants. I'm sure there are other potentials, but these are the ones that come to mind suddenly, never having had close contact with the specialty.”* P7

While there was some acknowledgment of the empirical and probabilistic risks associated with genetic conditions, this understanding was often superficial. Participants recognized the importance of discussing these risks, but many struggled to integrate this knowledge into a comprehensive counselling framework that addresses the multifaceted needs of patients.

*“Well, here, maybe even I need to understand a bit more about what can be done from the perspective of genetic counselling. Genetic counselling might not just be about doing tests and understanding mutations; it can also be about framing data, providing probabilities... even if that information is not very objective.”* P3

Some healthcare providers are unable to articulate what constitutes a GC session. This narrow perception overlooks the broader objectives and processes involved in GC, such as psychosocial support, management planning, patient education, ethical and legal considerations, communication skills, resource navigation, long-term follow-up, etc.

There is significant enthusiasm for the potential of ***genetics in personalised medicine***. Participants often viewed genetics as a tool for achieving definitive preventive care and treatment solutions, but the inherent complexities and interpretive challenges of genetic data suggest a need for more realistic and nuanced understanding. Participants expressed a particular interest in the potential of pharmacogenetics to optimise treatment. The potential for genetics to aid in preventive medicine was another highlighted area.

*"We can order tests and everything else, but it’s almost always to exclude other things and not to show that this disease exists, because that evaluation is based on what we see; on what the person tells us; and we deduce from that. So, I think it would be different and I think it would bring a certain objectivity to our practice that we still do not have. And that would be one of the goals of those who have been in this field for a long time. One of the desires is that we could base more on objective data and we are not yet doing that. Therefore, I think it would change the scope of the consultation somewhat if we integrated this genetic counselling."* P3

*“The identification of the problem in connection with a gene and its transmissibility has great benefits for disease prevention, or for being vigilant in other situations, or determining some risk situations that require additional care for that family or that syndrome."* P6

The ***current practices and management of PDs*** were also an emerged sub-theme. Participants recognized the subjective and multifactorial nature of PDs, emphasising the need for more scientific evidence in applying genetics to these disorders. They also noted the limited availability of genetic tests specific to PDs. Nevertheless, most of them did acknowledged the relevance of genetics in their clinical practice, with most of them engaging in family history risk assessments.

*“I think, therefore, my personal opinion is that in terms of psychiatric disorders with... with genetic detection or genetic screening, I believe that situation is not yet well clarified... because I don’t have any case on my list of a psychiatric disorder identified by genetic risks, although they may exist. I know they exist... therefore, in a family context, yes. With an associated gene, I don't believe the genetics consultation works well in that sense, but there is a family risk...”* P10

There is also an acknowledgement that more research is needed to establish clear genetic associations in psychiatric disorders.

*“But also, from what I know, there is no gene that is evidently associated. So, I think we still have a lot to discover. Based on the scientific evidence we have, for example, in oncogenetics, familial polyposis, BRCA, cardiomyopathies... that is already studied, we have this evidence, don’t we? Therefore, in the psychiatric part, perhaps due to my own misinformation... But it ends up being a bit difficult...”* P8

*“We have this scientific knowledge, but it’s not something we integrate into our daily practice, is it... we know that schizophrenia has familial aggregation, but if we have a schizophrenic patient or a suspect, a family member, we’re not going to do a test to diagnose. The diagnosis is still essentially clinical.”* P3

### Theme 2: barriers to PGC implementation

Within the second theme, three sub-themes emerged. Participants identified several ***biopsychosocial and cultural factors*** that act as barriers to the implementation of PGC. These included the stigma associated with PDs, and a general lack of sensitivity towards GC.

*“The issue of stigma... I think it influences. When I say stigma, I mean from patients, their families, and other healthcare professionals, okay? Because psychiatry is still seen by some colleagues from other specialties as something strange, ‘it’s all in their head’. They don’t view it as a disease; they think it’s just alterations…”* P1

Other contributing factors included concerns about the personal utility of genetic information, and resistance to changing existing practice models.

*“Psychiatric disorders are not necessarily considered like other diseases, so the problem starts there. But yes, I think genetic counselling in psychiatry is very important. Now, what’s up to us is to inform the population so they find it important.”* P9

*“Changing practices is always a barrier but also a potential, because once doctors see that their patients benefit from counselling, they naturally begin to adhere much more to the intervention. (…) There is a risk that no one will be referred, at least initially, until the habit is established”* P7

The variability in responses regarding patient inquiries about genetics suggests that while patients may occasionally be interested in genetic aspects, the overall engagement with GC remains limited.

***Practical challenges and applicability*** were another significant barrier. Issues such as resource management, funding, organisation, and accessibility were frequently mentioned.

*"Well, if it were just genetic counselling where we are not going to do anything, perhaps I would leave the consultation for those cases where we can actually do something... but that, perhaps, would depend more on your side... as a matter of resource management."* P5

*"Given that, so far, the world in quotes advances without any of this... probably the funding. Ah... I don’t know if it would be a priority, it would probably need to prove significant economic benefits to be funded."* P7

There were also concerns about the clinical utility of GC in psychiatric practice, particularly regarding how it could be integrated effectively into current healthcare systems.

*"Although we know that there is a very important genetic component, they do not require genetic counselling to the extent that they have an incidence determined by many other factors and, therefore, predictability is not possible. The phenotypes are not predictable, you know..."* P2

The centralization of genetic services leads to accessibility issues, particularly for patients in remote or underserved areas.

*"I also think one issue is the coordination with the (genetics) services for certain psychiatric services that are isolated, you know? Even if they are part of general hospitals, sometimes they are in peripheral buildings, you know? So, there is a certain distance."* P1

The ***need for education and guidelines*** was a prominent sub-theme. Participants expressed a desire for more training opportunities specifically tailored to their needs, indicating a gap in current educational offerings in genetics.

*“So, we read things, but there isn’t really… We don’t do internships in these areas. I mean, maybe some have done them, but it’s not very common, nor is it an area integrated into our training programme.”* P3

*"I can add that I would like there to be more training opportunities in genetics. The few I saw directed at family doctors, when I opened the programme, I thought it was not very focused on our daily practice."* P11

There is a perceived gap in the integration of genetic aspects within psychiatric practice, with participants noting that genetics is not often a primary focus in their daily clinical activities. They identified time constraints and a lack of awareness as possible factors delaying this integration. Nevertheless, they all agreed on the necessity for reliable information sources and the harmonisation of practice to ensure consistent and effective implementation of PGC.

*"And, we, family doctors are constantly updating... every day new information comes out... and the psychiatry part, it gets a bit sidelined. This is without a doubt... and sometimes it is a bit difficult for us to manage this in the consultation."* P11

*"In my daily clinical practice, I do not think much about the genetic aspect of pathologies, in psychiatric pathology. No, I won't say I do, because I don't... I don't think about it."* P8

### Theme 3: potentialities of PGC implementation

The third theme focused on the perceived benefits of implementing PGC, with three sub-themes emerging. Respondents underscored the value of a ***holistic and humanistic approach*** to care. They noted that person-centred care, which includes GC, could help demystify and destigmatise PDs, fostering a supportive environment for patients.

*"I think above all, it should have a more humanistic, empathetic, and destigmatising component. Because the idea of being able to, I imagine it is the same for any disease, but especially when we talk about psychiatric illnesses which already have so much stigma, I think some reassurance and empathy in handling these cases is crucial."* P4

Another key benefit highlighted was the ***power and value of information*** that may be provided through GC. Participants emphasised that such information could enrich the decision-making process, empower and educate patients, and uphold the patients' right to receive pertinent information in an appropriate manner.

*"For some people, it may be important to know. 'If I have a child, what will the risk be?' I believe people care deeply about this knowledge because they do not want their children to endure the same suffering.”* P1

*"Genetic counselling, in any area, and what I know best now is in oncology, is not about telling the person 'you have this and this will happen'. No. It provides information, in a context, in a certain way, which then says certain things, and eventually in the future... but it is always a probabilistic basis and therefore, in that sense, genetic counselling still makes sense, yes."* P3

*"I think it has implications both diagnostic and therapeutic, as well as in the information and psycho-education we do with patients about the fears they have."* P4

Finally, the potential of GC to enhance ***personalised and preventive medicine*** was again mentioned. Respondents pointed out the possibilities for risk stratification, the application of pharmacogenetics, and the implementation of preventive measures as significant advantages of integrating genetic insights into psychiatric care.

*"I think it could optimise treatment because perhaps if we had genetic data, we could use drugs without so much trial and error, right? If we know someone is a slow metaboliser, a fast metaboliser... If we had this information upfront, we could probably more easily adjust treatment plans."* P3

*"The potentialities, I think, depend on what the technique allows, right? That is, if it were possible for us to take a child and with genetic evaluation, we could predict with some degree of reliability what might happen in the future, that would be a great potential, not only for us to keep informed, for people to be aware, to have regular check-ups to prevent as early as possible."* P6

*"Also doing research, particularly in the family, depending on the type of situation we have, most of the time they are autosomal recessive, and therefore, the type of counselling to the family, screening parents and siblings, and therefore the counselling ends up being in the sense of prevention, regarding risk situations that this genetic condition might determine."* P2

## Discussion

This study provides a pioneering exploration of how family physicians and psychiatrists in Portugal perceive the provision of Genetic Counselling for Psychiatric Conditions. This is an emergent field of practice of GC, but a concept not yet established in our country. We sought to understand the perspectives of these key professionals on psychiatric genetics and GC within psychiatric context, acknowledging the growing global importance of this field. By employing an exploratory qualitative framework, the study provides some first insights and hopefully sets the stage for future discussions and research in this developing field.

Similarly to results found elsewhere (Finn et al. 2005; Zhou et al. 2014), our data suggests that psychiatrists and family physicians view genetic information as clinically relevant but have limitations of knowledge that may impact the incorporation of psychiatric genetics into clinical practice. Many interviewees found challenging to fully grasp the broad objectives and benefits of GC interventions. The association of GC primarily with the provision of information about genetic testing and recurrence risks was common, while relevant tasks and goals of GC consultations were largely unknown. Given that genetic testing for PDs is not routinely available in clinical practice, the immediate demand for GC in this area may be perceived as low. Unsurprisingly, the availability and mainstreaming of genetic testing seem to influence how GC is perceived and utilized.

A recent study examining the major obstacles to implementing PGC and genetic testing across European countries revealed that professionals identified the lack of guidelines and education as significant barriers (Koido et al. 2023). Our findings echoed these concerns, with interviewed professionals emphasizing the need for specialized training and clearer guidelines on how to integrate genetic knowledge into their practice. As genetic medicine becomes more integrated into primary and secondary care, the urgency for physicians to develop genetic literacy is growing. Although the importance of genetics is increasingly recognized, particularly with the advent of personalized medicine, the integration of this field into medical education is likely uneven across training institutions in the country. Assessing the extent and depth of genetic content in medical curricula and establishing clinical competencies in genetics and genomics, tailored to different professional backgrounds, would be valuable steps towards aligning Portuguese medical education more closely with European standards (ESHG 2009). To address these perceveid barriers, we suggest that integrating targeted education on genetic counselling and PGC into medical curricula and continuous professional development for psychiatrists and family physicians could be beneficial. Additionally, the development of clear, accessible guidelines delineating healthcare providers' roles and referral pathways in the context of PGC may help support more effective implementation.

Participants in this study highlighted the need for a holistic and humanistic approach in a potential PGC service, fostering a supportive environment for patients. Like other sub-specializations within GC, PGC requires additional professional competencies, particularly in tailoring the counselling process to address the unique emotional and psychological complexities of PDs. Stigma surrounding mental illness remains “the main obstacle to the provision of care for people with mental disorders” (Bennett et al. 2008; Sartorius 2007). Participants in this study were acutely aware of this challenge, recognizing its perpetuation not only among patients and their families but also within the healthcare community. This underscores the persistence of the issue and the importance of taking it into consideration. The authors emphasise the importance of careful communication to avoid reinforcing stigma or promoting a deterministic view of mental health. Austin (2020) provides a comprehensive 'Manual for Psychiatric Genetic Counselling,' serving as an essential professional resource to ensure that PGC is evidence-based and appropriately adapted to this context.

In Portugal, most genetic services are integrated into tertiary care located in major cities like Lisbon, Porto, and Coimbra, necessitating referrals to centralised services and creating some barriers to access (Costa et al. 2022). In our study participants highlighted the practical challenges of coordinating care across distances, leading to delayed or fragmented service provision in this specific context. At a systemic level, integrating PGC would require significant investment in training, capacity building, and resource allocation, potentially straining existing healthcare systems. While these specific considerations do not directly arise from our findings, they underscore the importance of careful planning and further investigation.

The creation and maintenance of multidisciplinary relationships and collaborations is crucial to ensuring high-quality healthcare (Costa et al. 2024a, b). We believe this is equally important in the context of GC for PDs. The implementation of PGC should be rooted in a multidisciplinary approach, involving, for instances, psychiatrists and primary healthcare providers working closely with medical geneticists, through joint consultations, referral systems, and case discussions. Adopting continuous and collaborative care could help address the current issues of fragmentation, while also considering resource limitations.

The recognition of genetic counsellors’ profession and their integration into the Portuguese healthcare could enhance the availability of genetic professionals and support the provision of comprehensive care, as for instance in the often-overlooked field of PDs (Slomp et al. 2022). Genetic counsellors, with specialized skills and training, are well-positioned to address these gaps. However, the lack of formal recognition of the profession in the country limits their ability to contribute effectively and delays the expansion of their roles into new areas and sub-specialties where their expertise can be an added value (Lungu et al. 2024). Additionally, recent national discussions among experts have pointed out the need for reforming medical genetics services and education, to better prepare future healthcare professionals for the challenges of integrating genetics into routine clinical care (Costa et al. 2024a, b).

Assessing the readiness for the implementation of PGC is indeed a complex task, and our data alone cannot provide a comprehensive evaluation. However, it appears that Portugal is still navigating certain fundamental challenges in genetic healthcare provision, which may indicate that the healthcare system might not yet be fully equipped to invest in sub-specialized services like PGC. While the enthusiasm among psychiatrists and family physicians to learn and have PGC alongside their care practice is encouraging, the necessary groundwork seems to have not yet been laid. It will require a concerted effort across multiple domains, including education, policy, and service delivery. By continuing to explore and address these challenges, the path toward effective and widespread implementation of PGC in Portugal can become clearer and more attainable.

### Study limitations

One of the limitations of this study is the potential self-selection bias among participants. Those who volunteer to participate may already have a heightened sensitivity or interest in the topics of genetics and genetic counselling. As a result, their views and attitudes may not be fully representative of the broader population of psychiatrist and family physicians in the country.

Response bias is another potential limitation of this study. Participants might have provided answers they deemed more socially acceptable or expected, rather than their true beliefs and attitudes. This could affect the authenticity and reliability of the findings.

### Further research on this topic

As for the next steps in research, a more comprehensive evaluation of the readiness for PGC implementation is essential. Future studies should aim to include the perspectives of medical geneticists, who are crucial stakeholders in this field. Additionally, exploring the needs of patients, particularly those who might benefit most from PGC, could provide valuable insights into how services should be structured.

Demographic factors such as age and years of experience may influence perceptions of the genetic counselling practice, as they are often linked to differences in training and exposure to genetic counselling; however, the data from this study do not allow for definitive conclusions on this matter. In future studies with larger and more diverse samples, it would be interesting to explore this, as well as incorporating additional demographic and contextual factors such as workplace setting and prior involvement with genetic services to better understand how these characteristics shape attitudes towards genetic counselling.

Evaluating the depth and breadth of genetic content in medical curricula, alongside establishing clinical competencies tailored to various healthcare backgrounds, will be essential for aligning Portuguese medical education with European standards. Moreover, policy analysis will be vital in identifying both enablers and barriers within the healthcare system that could impact the successful implementation of PGC. By addressing these aspects, future research can support the development of a robust, patient-centered approach to PGC in Portugal.

## Conclusion

This study offers an initial exploration into the perceptions of family physicians and psychiatrists in Portugal regarding the role of genetics and the potential for GC in the context of psychiatric illnesses. Results show that while the importance of genetics in PDs is acknowledged, there is low literacy about GC practice from the professional groups interviewed. The availability and mainstreaming of genetic testing seem to influence how GC is perceived and utilized. Additionally, there is a perceived need for training and guidelines that foster the dissemination of genetics into healthcare, specifically mental healthcare. A holistic and patient-centred approach is considered essential in managing PDs and, by extension, in PGC, as it addresses both medical and psychosocial factors. Although psychiatrists and family physicians are keen to integrate PGC into their patients’ care, it seems that certain fundamental challenges persist in genetic healthcare provision.

## Data Availability

No datasets were generated or analysed during the current study.
